# Simulating porcelain firing effect on the structure, corrosion and mechanical properties of Co–Cr–Mo dental alloy fabricated by soft milling

**DOI:** 10.1007/s10266-023-00849-2

**Published:** 2023-08-29

**Authors:** Angeliki G. Lekatou, Sevasti Emmanouilidou, Konstantinos Dimitriadis, Maria Baikousi, Michael A. Karakassides, Simeon Agathopoulos

**Affiliations:** 1 Department of Materials Science and Engineering, School of Engineering, University of Ioannina, 451 10, Ioannina, Greece; 2 Institute of Materials Science and Computing, University Research Center of Ioannina (URCI), 451 10, Ioannina, Greece; 3 Division of Dental Technology, Department of Biomedical Sciences, University of West Attica, 122 43, Athens, Greece

**Keywords:** Co–Cr–Mo dental alloy, Soft milling, Corrosion, Nanoindentation, Mechanical properties

## Abstract

This study aims at evaluating the effect of simulating porcelain firing on the microstructure, corrosion behavior and mechanical properties of a Co–Cr–Mo alloy fabricated by Metal Soft Milling (MSM). Two groups of Co-28Cr-5Mo specimens (25 × 20 × 3 mm) were prepared by MSM: The as-sintered (AS) specimens and the post-fired (PF) specimens that were subjected to 5 simulating porcelain firing cycles without applying the ceramic mass onto their surface. Phase identification by X-ray Diffraction (XRD), microstructure examination by optical microscopy and Scanning Electron Microscopy combined with Energy-Dispersive X-ray Spectroscopy (SEM/EDX), corrosion testing by cyclic polarization and chronoamperometry in simulated body fluid (SBF), the latter test accompanied by Cr^3+^ and Cr^6+^ detection in the electrolyte through the 1.5-diphenylcarbazide (DPC) method and UV/visible spectrophotometry, and mechanical testing by micro-/nano-indentation were conducted to evaluate the effect of the post-firing cycles on the properties of Co–Cr–Mo. The results were statistically analyzed by the t test (p < 0.05: statistically significant). All specimens had a mixed γ-fcc and ε-hcp cobalt-based microstructure with a dispersion of pores filled with SiO_2_ and a fine M_23_C_6_ intergranular presence. PF led to an increase in the ε-Co content and slight grain coarsening. Both AS and PF alloys showed high resistance to general and localized corrosion, whereas neither Cr^6+^ nor Cr^3+^ were detected during the passivity-breakdown stage. PF improved the mechanical properties of the AS-alloy, especially the indentation modulus and true hardness (statistically significant differences: p = 0.0009 and 0.006, respectively). MSM and MSM/simulating-porcelain firing have been proven trustworthy fabrication methods of Co–Cr–Mo substrates for metal-ceramic prostheses. Moreover, the post-firing cycles improve the mechanical behavior of Co–Cr–Mo, which is vital under the dynamically changing loads in the oral cavity, whereas they do not degrade the corrosion performance.

## Introduction

Metals and their alloys, which constitute a major group of biomaterials, are widely used in dentistry for many years [1]. In dental prosthetics, cobalt–chromium alloys (Co–Cr) are preferred instead of nickel–chromium alloys (Ni–Cr) for removable dental frameworks and metal-ceramic restorations because nickel allergies are a relatively common problem [1, 2]. Molybdenum (Mo) is commonly added as an alloying element to increase the hardness and strength of the alloys by solid solution strengthening and decreasing the stacking fault energy, thereby hindering dislocation climbing and gliding [3, 4]. Dental restorations are conventionally fabricated using traditional lost-wax casting (Cast) techniques because of equipment simplicity and low cost. Nonetheless, metal shrinkage may occur, causing formation of pores or defects in the cast metal during solidification [5].

However, the recent technological developments in computer aided design and computer aided manufacturing (CAD/CAM) processes have boosted the production of Co–Cr–Mo dental prosthetic restorations by additive or subtractive manufacturing processes [5–7]. More specifically, nowadays, the metal parts of metal-ceramic prosthetic restorations can be produced using three different CAD/CAM-based technologies: hard milling (HM, which is a subtractive technique), selective laser melting (SLM, which is an additive manufacturing technique) and milling of soft metal, also called soft milling (MSM, which is a subtractive technique) [5]. The first two techniques (HM, SLM) lead to high-quality dental restorations, i.e., free of porosity, with values of mechanical properties, electrochemical properties and metal-ceramic bond strength that satisfy the specifications for metal-ceramic restorations [5–9]. Nonetheless, they present some limitations. For instance, ΗΜ of Co–Cr blanks may be difficult because of the hardness of the solid blank and the consequent demands on the manufacturing equipment (coolant delivery, rigidity of the machine, fast tool wear, high maintenance costs etc.) [10, 11]. On the other hand, SLM also presents limitations, among which there is a risk of powder evaporation by high-power laser beams and difficulty in removing unmelted powder particles from fabricated parts [12].

An alternative manufacturing process to HM and SLM, is the recently developed MSM method. This technique includes dry milling of green Co–Cr blocks, where unsintered metal powder particles are held together by an organic binder, being, thereby, easy to be processed in the preliminary state. Following milling from the blank, the frameworks are densely sintered in a downstream process. The milled Co–Cr structures are sintered at approximately 1300–1350 °C under a protective gas atmosphere. During the sintering process, the organic binder is eliminated by evaporation, and the metallic powder particles are sintered to a dense body through a volume shrinkage of ~ 10% [11, 13–15]. As a result, homogeneous and distortion-free frameworks without contraction cavities are claimed to be produced [11]. Nowadays, the standardized industrial conditions for producing predesigned restorations by MSM may ensure a reduced milling time [16]. Moreover, the similarity of the processing steps to those of pre-sintered ZrO_2_, allows the application of the technique in ordinary dental laboratories that employ CAD/CAM equipment [17].

In metal-ceramic prosthetic restorations, problems usually result from biological complications (primarily due to poor marginal or internal fit leading to plaque accumulation), electrochemical complications (poor corrosion resistance) and mechanical complications [1, 18]. The mechanical properties and corrosion resistance of the Co–Cr alloys are closely related to their microstructure, which depends on the chemical composition, manufacturing technique and heat-treatment process [6].

The manufacturing process of metal-ceramic prosthetic restorations involves a porcelain firing procedure that includes both high temperatures and repeated firing cycles (between 900 and 1000 °C) according to the instructions of the ceramic material manufacturer.

Recently, several research teams evaluated the physical and mechanical properties, as well as the corrosion resistance of MSM Co–Cr. Regarding the corrosion resistance, Rylska et al. [19] reported that among Co–Cr alloys subjected to a porcelain firing procedure, the SLM alloy exhibited the highest corrosion resistance in 0.9 wt.% NaCl, followed by the MSM and the cast alloy. A soft-milled Co–Cr alloy showed a significantly lower Co-ion release than that of its investment cast analogue [17]. Reclaru et al. [11] compared the corrosion performance (in artificial saliva) of Co–Cr–Mo manufactured by five different techniques: CAD/CAM milling, Direct Metal Laser Sintering, Selective Laser Melting, Electron Beam Melting, and CAD/CAM milling followed by sintering; regarding the latter technique, they noted that the presence of binder and the final sintering process may lead to increased corrosion of the prosthetic milled piece.

Regarding the mechanical performance, both laser sintered and soft-milled Co–Cr alloys were found suitable for long-lasting metallic prosthodontic frameworks; the 3D-printed alloy exhibited superior tensile properties, likely due to lack of porosity [20]. Hong et al. demonstrated the paramount importance of the crystal structure and microstructure on the tensile strength of various commercially available soft metal milling blanks and systems [10]. According to Al Jabbari et al. [5], SLM leads to the highest hardness, followed by HM, Cast, and MSM; SLM manifested the highest elastic modulus, followed by MSM, HM and Cast. Kim et al. [21] ranked four manufacturing techniques of Co–Cr–W alloys in decreasing order of mechanical performance: Selective laser melting, soft milling, casting, milling. Soft-milled Co–Cr showed substantially greater elongation, whereas Co–Cr fabricated by rapid prototyping (SLM) exhibited higher proof strength compared to conventionally cast Co–Cr [22].

There is limited documentation on the effect of consecutive porcelain firing cycles, such as those employed in the fabrication of metal-ceramic restorations, on the microstructure, corrosion resistance and mechanical properties of dental Co–Cr–Mo fabricated by MSM. Rylska et al. [19] applied a a full porcelain firing schedule on SLM, MSM and Cast Co–Cr, and evaluated the corrosion resistance of the heat-treated alloys, as aforementioned. Önöral et al. [15] conducted repeated firings on Co–Cr alloys fabricated by casting, fully sintered hard alloy milling (FHAM), presintered soft alloy milling (PSAM) and selective laser sintering (SLS); the PSAM-fabricated restorations exhibited the greatest fitting accuracy. Ogunc and Avcu [23] applied repeated firings on Co–Cr alloys prepared by casting, selective laser sintering (SLS), presintered soft metal milling (PSMM) and post-sintering hard metal milling (PHMM). After the firing cycles, the PSMM and SLS alloys had better marginal and internal adaptation relatively to the casting and PHMM groups. Kocaağaoğlu et al. [18] concluded that the laser sintered, and soft-milled Co–Cr alloys demonstrate better marginal adaptation compared to the lost-wax group after repeated ceramic firings.

Therefore, this in-vitro study reports on this subject. More specifically, the null hypothesis (H_o_) is that no significant alterations occur in the Co–Cr–Mo alloy after a simulating porcelain firing procedure. In other words, the simulating porcelain firing procedure does not jeopardize the microstructure, corrosion behavior and mechanical properties of a Co–Cr–Mo dental alloy fabricated by MSM and, subsequently, the expected clinical performance of the Co–Cr–Mo/porcelain restoration.

## Materials and methods

Eight plates of a Co–Cr–Mo dental alloy (Ceramill Sintron; Amann Girrbach, Koblach, Austria) of a nominal composition of Co 66, Cr 28, Mo 5, Si < 1, Fe < 1, Mn < 1 ((in wt.%) and dimensions of 25 × 20 × 3 mm, were prepared using a CAD software (Siemens NX 12) and a Ceramill Motion 2 milling machine (Amann Girrbach, Koblach, Austria). The specific dimensions were chosen so as to achieve the desired dimensions after metallographic preparation of the specimens (grinding and polishing procedures) as recommended by ISO 10271 [24]. After the MSM procedure, the green (non-presintered) Co–Cr–Mo specimens were subjected to sintering at 1350 °C in a high-temperature furnace filled with Ar gas. The produced Co–Cr–Mo specimens were equally divided into two groups. The first group included the as-sintered (AS) plates. The second group included the porcelain-fired (PF) plates that had been subjected to 5 simulating porcelain firing cycles (Table 1) in a special oven (P510 Programat; Ivoclar Vivadent, Ellwangen, Germany) according to the manufacturer’s instructions (namely the Company Vita, VMK-Master, Vita, Bad Sackingen, Germany), without applying the ceramic mass onto their surface. The above Co–Cr–Mo specimens were evaluated by the following techniques:

**Table 1:** Conditions of the simulating porcelain firing procedure according to the manufacturer’s instructions (Company Vita, VMK-Master, Vita, Bad Sackingen, Germany), applied in the present work to the specimens of the PF group

Simulating porcelain firing procedure	Low temperature (dry-preheat time)	High temperature (heating rate, holding time, atmosphere)
Bonding agent fire	600 °C (5–7 min)	960 °C (60 K/min, 1 min, air)
Opaque fire	500 °C (5–7 min)	950 °C (80 K/min, 1 min, air)
Dentine 1 fire	500 °C (5–7 min)	930 °C (55 K/min, 1 min, air)
Dentine 2/Enamel fire	500 °C (5–7 min)	920 °C (55 K/min, 1 min, air)
Glaze fire	500 °C (5–7 min)	920 °C (80 K/min, 1 min, air)

Phase identification was carried out by X-ray diffraction (XRD) analysis using the Bruker D8 Advance diffractometer (Bruker AXS; Billerica, MA, USA) under the following parameters: Ni-filtered Cu K_α_ radiation (λ = 1.5406 Å); 2θ range of 20–100°; standard slit; step size of 0.004°/s.

The weight percentage of the εCo phase in the alloys was calculated from the intensities (I) of the XRD peaks (101)ε and (220)γ based on the equation originally developed by Sage and Gillaud [25]:

1 $$wt. \% \left(hcp\right)=\frac{{I}_{\left(101\right)\varepsilon }}{{I}_{\left(101\right)\varepsilon }+1.5 {I}_{\left(200\right)\gamma }}$$

Microstructural examination was conducted by Scanning Electron Microscopy (SEM, JSM-6510 LV; JEOL; Japan) complemented with Energy-Dispersive X-ray spectroscopy (EDX, X-Act; Oxford Instruments; UK), under Secondary Electron (SE) mode and Backscattering Electron Composition (BEC) mode. Optical microscopy was also carried out (Leica 4000 DM; Leica Microsystems, Wetzlar, Germany). The metallographic preparation of the specimens included mounting in a phenolic resin (Presi, Eybens (Isère), France), grinding to 2400 grit by SiC papers and polishing to 1 μm by diamond sprays (Diamant Mecaprex Spray; Presi, Eybens (Isère), France) using the Ecomet 6 equipment (Buehler, Illinois Tool Works (ITW), Lake Bluff, IL, USA). The grain structure was revealed through electrochemically etching of polished surfaces (HCl/HNO_3_/FeCl_3_: 40 ml/1 ml/13 g, 6 V, 15 s, room temperature). Finally, the polished and etched Co–Cr–Mo specimens were rinsed with plenty of water, ultrasonically cleaned and blow-dried.

The grain size of the alloys was calculated on polished specimens (×500) using the ImageJ software (Wayne Rasband, National Institutes of Health, Bethesda, Maryland, MD, USA). The grain size of each alloy was extracted by averaging the measurements from 10 independent fields of view.

Polished surfaces of coupons of AS and PF specimens mounted in phenolic resin were peripherally covered by PTFE, leaving a surface area of about 1 cm^2^ to be exposed to a simulated body fluid (SBF) solution, at 37 °C. SBF had a composition of 7.996 g NaCl, 0.350 g NaHCO_3_, 0.224 g KCl, 0.228 g K_2_HPO_4_.3H_2_O, 0.305 g MgCl_2_·6H_2_O, 40 ml 1 N HCl, 0.278 g CaCl_2_, 0.071 g Na_2_SO_4_, 6.057 g NH_2_C(CH_2_OH)_3_, all in 1 l of bidistilled water, and a pH of 7.4 at 37 °C [26]. Potentiodynamic polarization tests were carried out connecting a standard three electrode cell (working electrode: Co–Cr–Mo surface, reference electrode: Ag/AgCl/3.5 M KCl, counter electrode: Pt gauze) to an ACM Gill AC galvanostat/potentiostat (ACM Instruments, Cumbria, UK). After 1 h holding under open circuit to achieve steady-state, potentiodynamic polarization started at a scan rate of 10 mV/min. The corrosion current densities (i_corr_) were determined by Tafel extrapolation on the cathodic part of the polarization curves by linear regression analysis of the potential against the logarithm of current density (log (i)) data. The reliability of the calculations was ensured by conforming to several restrictions, detailed in previous studies [27, 28]. The resistance of the alloys to forms of localized corrosion was assessed by cyclic polarization. The basic concept of this technique is that the exposed surface will undergo a form of localized corrosion if the current density of the reverse anodic scan is greater than the current density of the forward anodic scan for the same potential, due to a more aggressive environment in the pits/crevices. In this case, a clockwise hysteresis loop is formed in the voltammogram [29, 30]. The corrosion rates were extracted from the i_corr_ values according to ASTM G102. Chronoamperometry testing (i.e., potentiostatic testing that records the variation of current density versus time of immersion in the electrolyte) was conducted in SBF at 37 °C, in order to validate the corrosion stages suggested by potentiodynamic anodic polarization.

The 1.5-diphenylcarbazide (DPC) method was employed to measure the Cr^6+^ and Cr^3+^ concentration in the solution after chronoamperometry testing. According to this method, when Cr^6+^ reacts with 1.5-diphenylcarbazide under acidic conditions, a purple-colored complex is formed, which, under a UV–Visible (UV–vis) spectrophotometer, exhibits an absorbance peak at λ = 540 nm [31]. In the present case, the solutions for the measurement were prepared by adding 60 μl of H_3_PO_4_ solution (0.5 ml of H_3_PO_4_ (85%) in 10 ml of H_2_O) and 120 μl of DPC solution (0.025 g DPC in 10 ml acetone) in 2.820 ml of the electrolytes after chronoamerometry testing of the alloys. The absorbance measurements were conducted in quartz cuvettes by a two-beam spectrophotometer (UV-2401(PC); Shimadzu; Kyoto, Japan) using a halogen lamp (range of 400–700 nm, step of 0.5 nm) with the aid of a calibration curve. The latter was created from different standard solutions of Cr^6+^ of known concentrations in the range of 0 − 1 mg/l). After the determination of Cr^6+^, Cr^3+^ detection in the electrolyte after chronoamperometry was performed by the reaction of 3.8 ml of the solution with 21 μl of KMnO_4_ solution (35 mg of KMnO_4_ in 10 ml of H_2_O), in order to ensure the oxidation of Cr^3+^ to Cr^6+^ [31]. In turn, the concentration of Cr^6+^ resulting from the oxidation of Cr^3+^ was determined using the 1,5-DPC method and UV/visible spectrophotometry.

The microhardness of the metallographically prepared Co–Cr–Mo specimens was measured according to ASTM E384 [32] using the HMV (Shimadzu, Kyoto, Japan) tester, under the parameters: load of 4.903 N (HV 0.5), dwell time of 15 s, 10 indentations per sample, 3 samples per state (AS or PF). The Co–Cr–Mo specimens were further tested by Instrumented Indentation Testing employing a nano-indentation testing machine (DUH-211S; Shimadzu Europa GmbH, Germany). The indentation modulus (Eit), indentation hardness (Hit) and true hardness (Hit/Eit) were determined under the parameters: standard Berkovich indenter, test depth of 500 nm, loading rate of 10 mN/s, 15 indentations per sample, 3 samples per state (AS or PF).

The statistical significance of differences between the mechanical properties of the groups was evaluated by the t test. In all tests, the statistical difference was considered significant only if the probability value (p) was ≤ 0.05. The normality and homoscedasticity were determined by the Shapiro Wilk and the equal test variance, respectively.

## Results

**Fig. 1:**
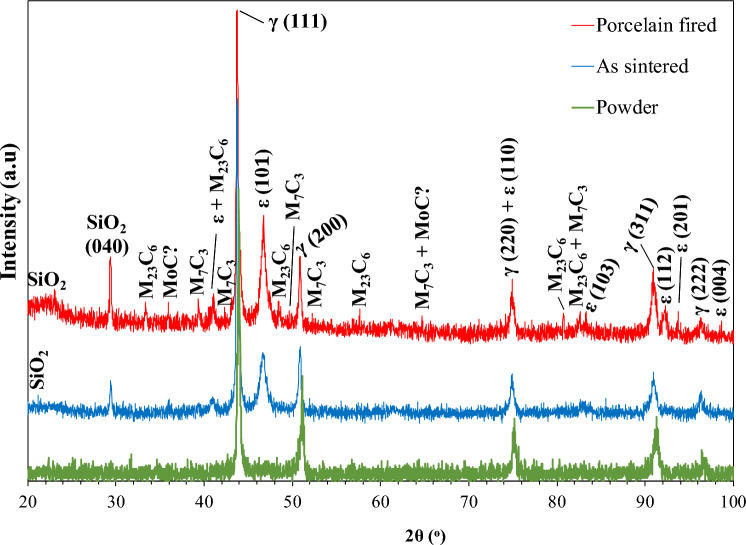
X-ray diffractograms of the powder, the AS and the PF Co–Cr–Mo alloy; γ: 15–806, ε: 5–727, M_23_C_6_: 35–783, M_7_C_3_: 36–1492, MoC: 45–1015, SiO_2_: 12–708

The X-ray diffractograms of the AS and PF Co–Cr–Mo specimens and the raw powder are plotted in Fig. 1. Figure 1 shows that the AS and PF Co–Cr–Mo alloys mainly consist of the two allotropes of Co, namely a Co-based phase of fcc crystal lattice (γ-Co or α-Co), which is stable at high temperatures, and a Co-based phase of hcp crystal lattice (ε-Co), which is stable at low temperatures [33]. Figure 1 indicates that the PF alloy has a greater martensite (ε-Co) presence compared to the AS alloy. Indeed, the ε-Co percentages in the AS alloy and PF alloy, determined by Eq. (1), were 37% and 45%, respectively. The carbide phases of M_23_C_6_, M_7_C_3_ (M: Cr, Mo) and possibly MC (M: Mo, Cr) have also been identified, the M_23_C_6_ stoichiometry appearing to be the predominant one (at least in the PF alloy). Higher amounts of carbides in the PF alloy compared with the AS alloy are implied by the higher intensities of the respective peaks. The XRD pattern also revealed the presence of SiO_2_. The XRD pattern of the Co–Cr–Mo raw powder shows only γ-fcc peaks, unlike the AS and PF Co–Cr–Mo specimens.

**Fig. 2:**
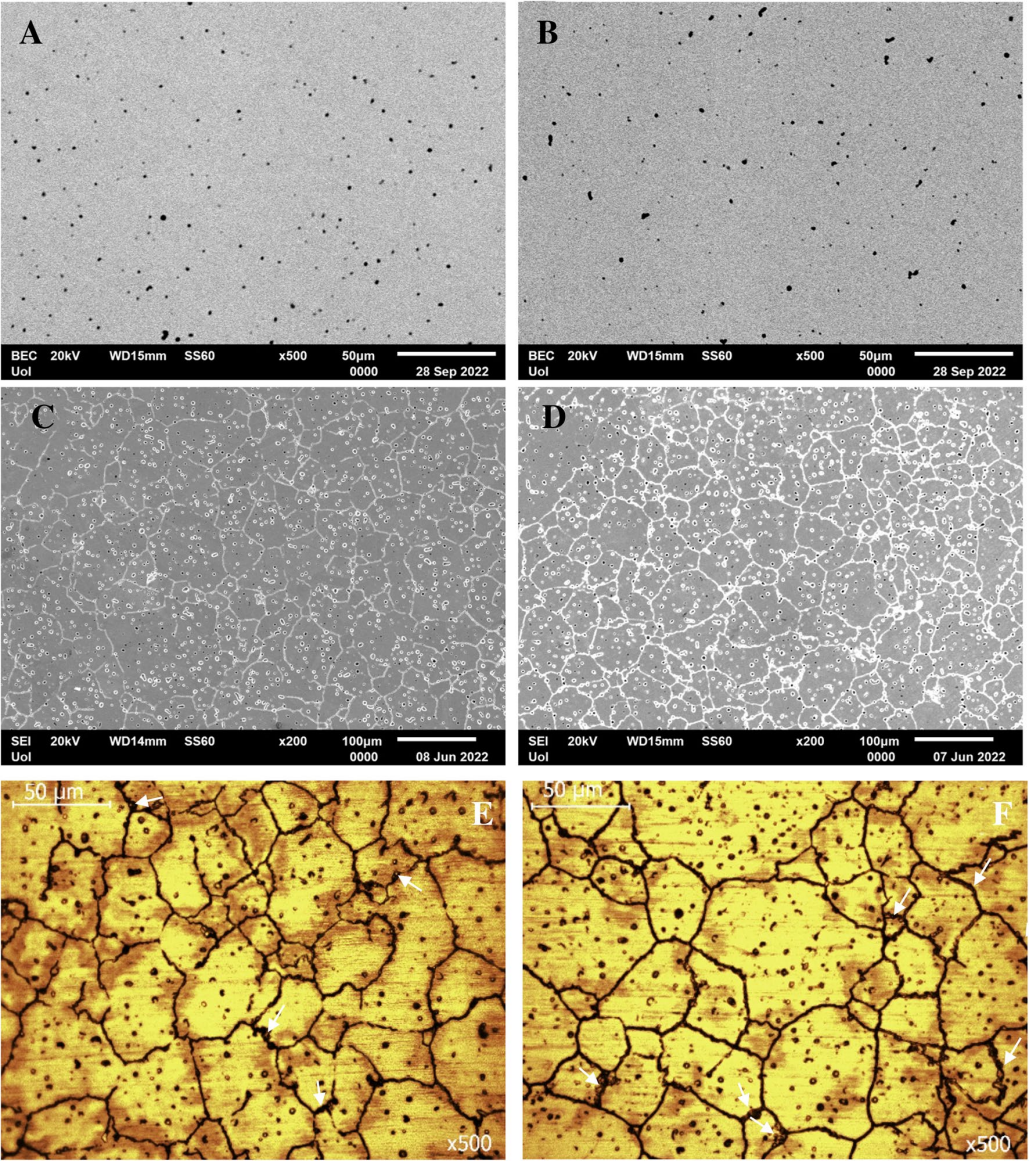
Microstructures of the AS (**A**, **C**, **E**) and PF (**B**, **D**, **F**) specimens before (**A**, **B**) and after electrochemical etching (**C**–**F**); (**A**, **B**): BEC mode, (**C**, **D**): SE mode, and (**E**, **F**): under optical microscope; the white arrows in (**E**, **F**) point at intergranular carbide particles

**Fig. 3:**
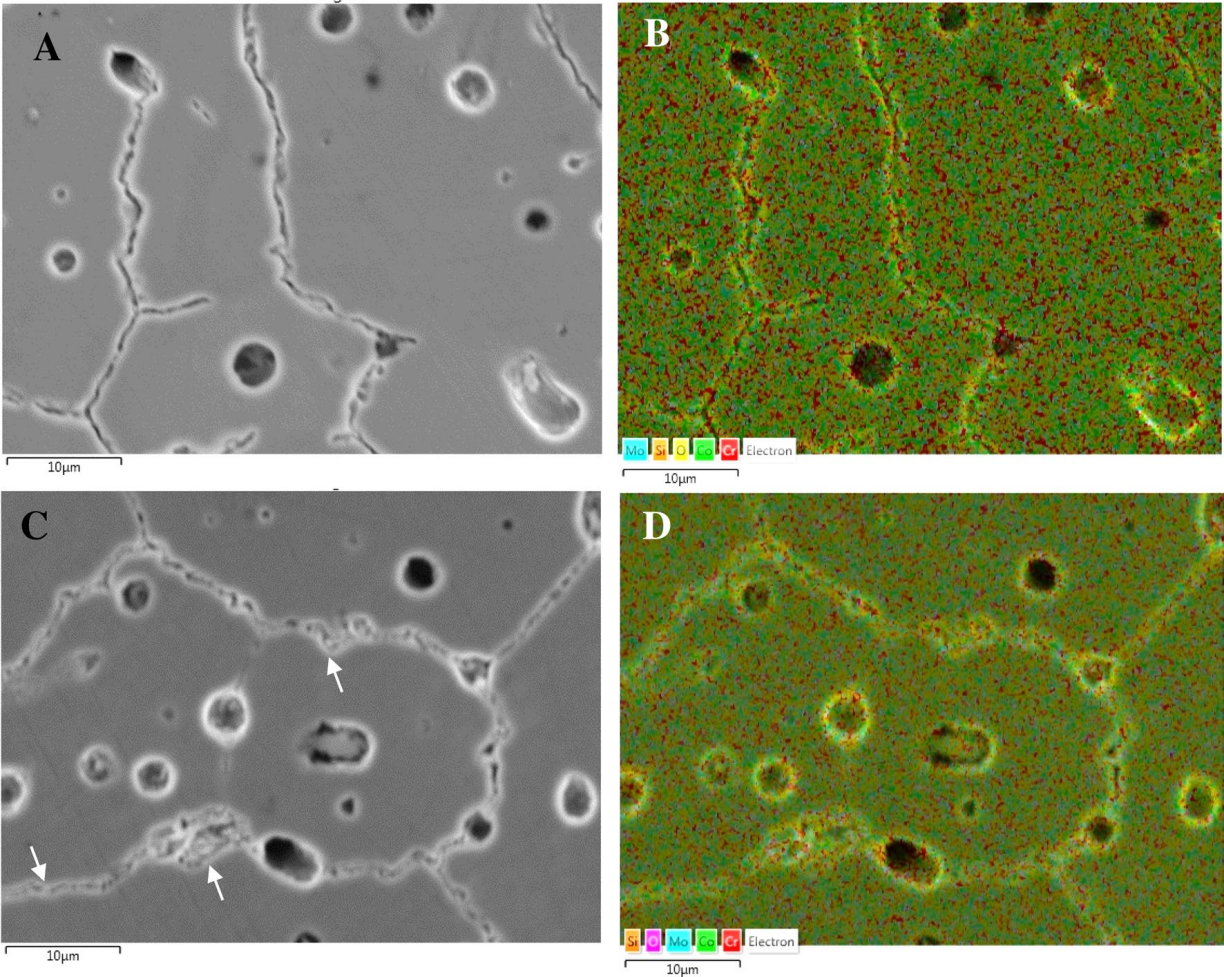
SEM-SE images of AS (**A**) and PF (**C**) specimens and respective EDX elemental mapping. **B** turquoise (Mo), orange (Si), yellow (O), light green (Co), red (Cr); **D** orange (Si), magenta (O), turquoise (Mo), light green (Co), red (Cr). The arrows point at carbide particles

The microstructures of the AS and PF Co–Cr–Mo specimens are presented in Fig. 2A–F. As seen in Fig. 2C–F, the grain boundaries in the Co–Cr–Mo specimens were clearly visible after electrochemical etching. Fine particle precipitation is discerned along grain boundaries, pointed at by white arrows in Fig. 2E, F. Both groups present similar microstructures. Nevertheless, on a closer observation, the PF alloy presents a slight grain coarsening; indeed, the grain size of the AS alloy and PF alloy was measured as 48 ± 16 μm and 64 ± 14 μm, respectively. In addition, the PF specimens manifest more intensively outlined grain boundaries in relation to the AS specimens despite the identical conditions of etching (compare Fig. 2C, D). On a larger magnification, the micrographs of Fig. 3 reveal precipitation of fine particles along the grain boundaries of the AS and PF specimens. The EDX maps in Fig. 3 show increased Mo and Cr concentrations along the grain boundaries. The grain boundaries of the PF alloy in Fig. 3C appear thicker than those of the AS alloy.

**Fig. 4:**
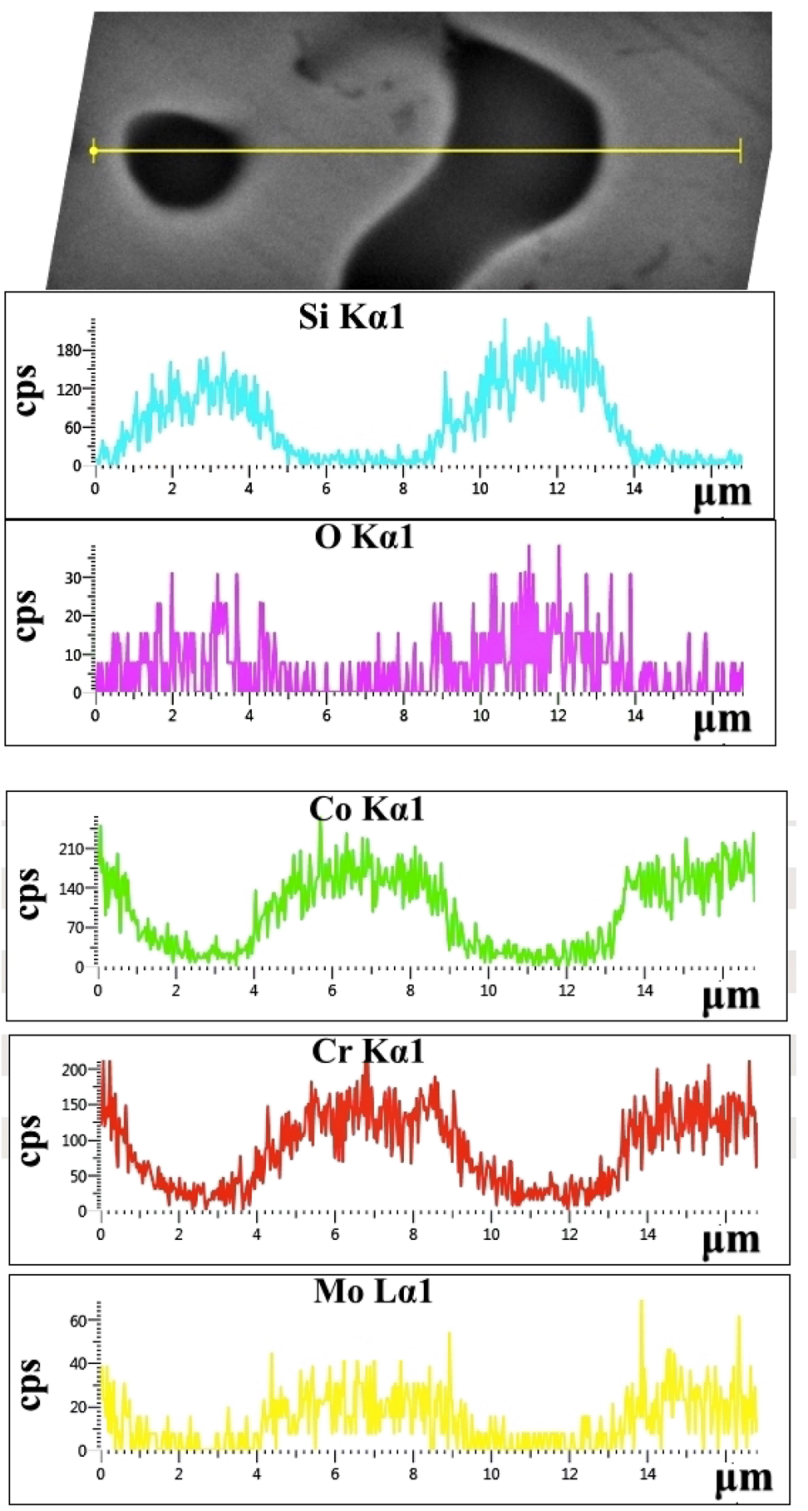
SiO_2_ particles in the AS alloy and EDX-line scanning for Si, O, Co, Cr and Mo

However, both groups exhibit a diffuse distribution of pores of diameter of the order of 2–3 μm (Fig. 2A–F, Fig. 3). According to the results of the EDX analysis (Fig. 4), many of the pores are filled with Si-oxide particles. This finding confirms the results of the XRD analysis. Figure 3 also shows that most of the pores are filled with Si-oxide.

**Fig. 5:**
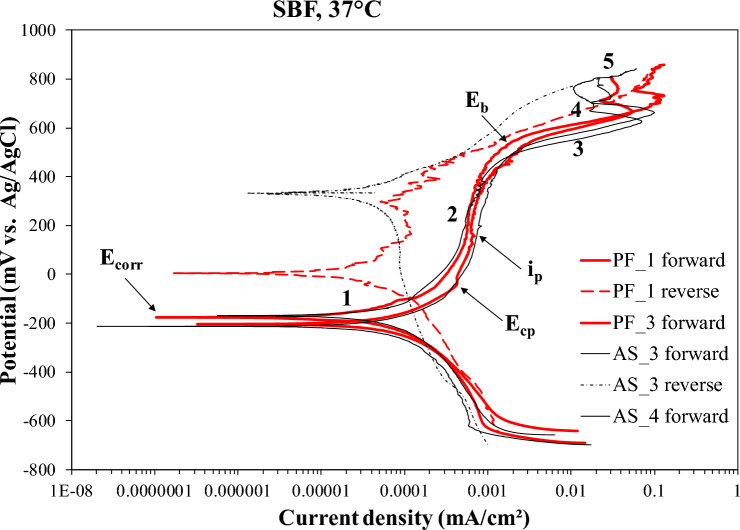
Cyclic polarization behavior of the AS and PF alloys (SBF, 37 °C). E_corr_, E_cp_, E_b_: corrosion potential, critical passivation potential, breakdown potential, respectively, i_p_: passive current (in the middle of the passive stage)

Figure 5 presents representative potentiodynamic polarization curves of the AS and PF Co–Cr–Mo alloys. In both groups, almost identical polarization curves were obtained with similar electrochemical values, the latter given in Table 2. Table 2 shows that the differences in the mean corrosion values of the AS and PF specimens fall within standard deviation. In both cases, very low corrosion current density (i_corr_) values and passive current density (i_p_) values have been attained. Abrupt changes in the anodic curve divide them in five stages, which are noted in Fig. 5 and will be discussed in the Discussion section. Figure 5 shows that the reverse current densities are lower than the forward current densities at the same anodic potential forming counterclockwise hysteresis loops of marked surface areas. However, it should be noted that a limited number of replicates showed clockwise hysteresis loops of low surface areas (AS: 2 out of 10 and PF: 3 out of 10).

**Table 2:** Corrosion values for AS and PF Co–Cr–Mo alloys (SBF, 37 °C)

Alloy	E_corr_ (mV, Ag/AgCl)	E_b_ (mV, Ag/AgCl)	i_corr_ (mA/cm^2^)	i_p_ (mA/cm^2^)	r_corr_ (mm/y)	Pit
AS	− 191 ± 31	514 ± 31	3.1 × 10^–4^ ± 0.8 × 10^–4^	8 × 10^–4^ ± 2 × 10^–4^	0.0030 ± 0.0008	No (8/10)
PF	− 190 ± 39	487 ± 23	2.1 × 10^–4^ ± 0.6 × 10^–4^	7 × 10^–4^ ± 1 × 10^–4^	0.0021 ± 0.0006	No (7/10)

*E*_*corr*_ corrosion potential, *E*_*b*_ breakdown potential, *i*_*corr*_ corrosion current density, *i*_*p*_ passive current density and *r*_*corr*_ corrosion rate

**Fig. 6:**
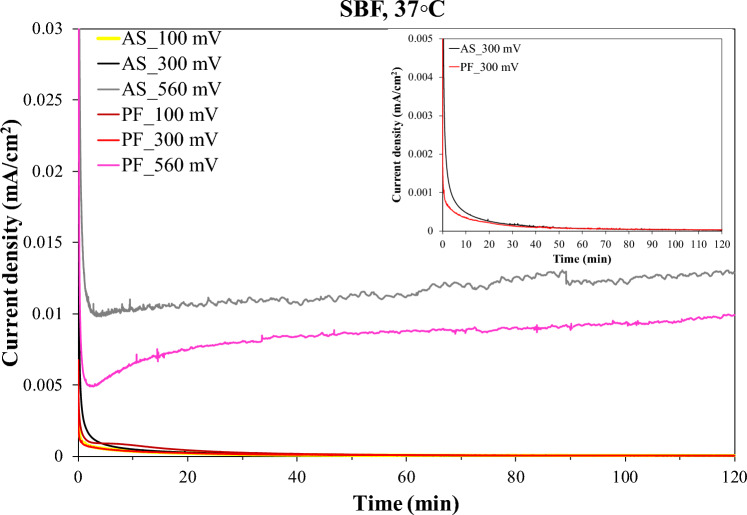
Cronoamperometry plots of the AS and PF alloys at anodic potentials in passive stage 2 (100 mV, 300 mV vs. Ag/AgCl) and breakdown stage 3 (560 mV vs. Ag/AgCl). Inset: chronomperometry plot at 300 mV vs. Ag/AgCl

Figure 6 includes the current density against time of immersion in SBF plots at potentials in stage 2 and stage 3, as noted in Fig. 5. Chronoamperometry recorded very low final currents in stage 2 (order of 10^–5^ mA/cm^2^) and higher final currents in stage 3, though still fairly low (order of 10^–2^ mA/cm^2^). The current density vs. time curves show very similar (practically the same) trends for both alloys but somewhat different trends between stage 2 and stage 3.

**Fig. 7:**
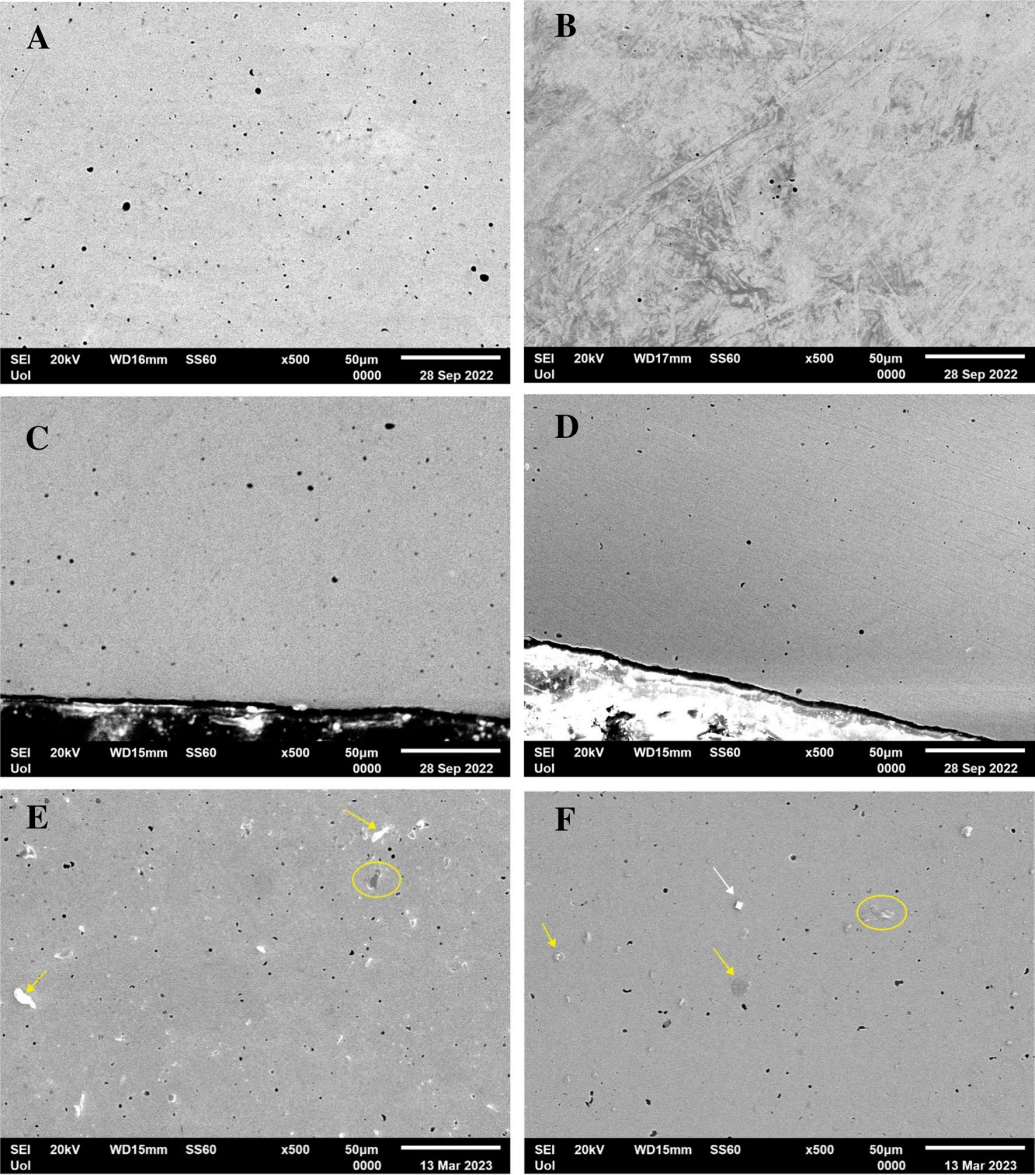
SEM micrographs of the alloys after cyclic polarization (**A**–**D**) and chronoamperometry (**E**: 560 mV vs Ag/AgCl, **F**: 300 mV vs. Ag/AgCl) in SBF, at 37 °C. **A**, **B**, **E**, **F** Surface view; **C**, **D** Cross-sectional view; **A**, **C**, **E** AS alloy; **B**, **D**, **F** PF alloy

**Fig. 8:**
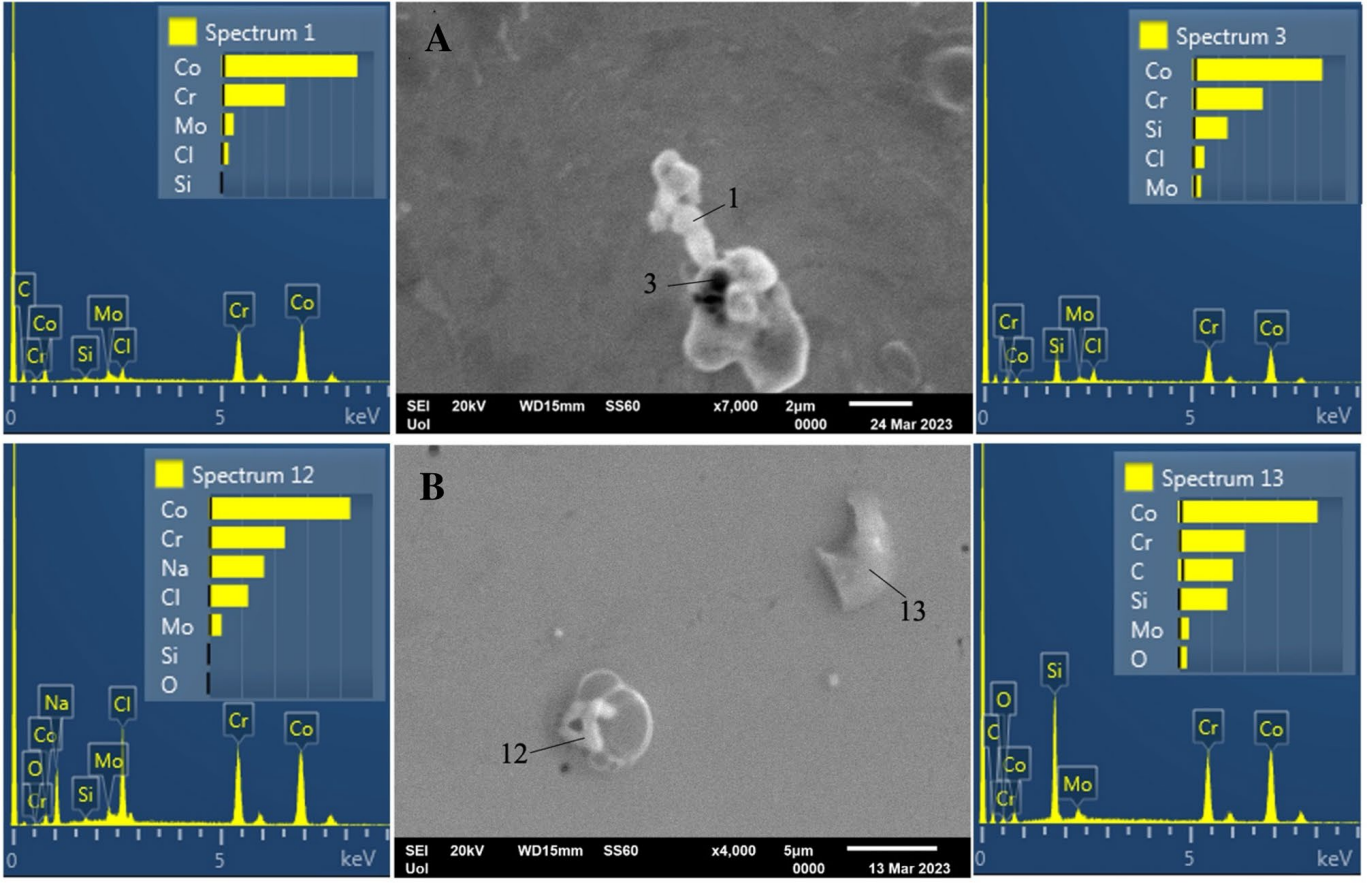
Surface depositions after chronoamperometry of: **A** AS alloy at 560 mV vs. AgAgCl (transpassive s tage 3), and **B** PF alloy at 300 mV vs. Ag/AgCl (passive s tage 2), in SBF, at 37 °C, and respective EDX point spectra

**Fig. 9:**
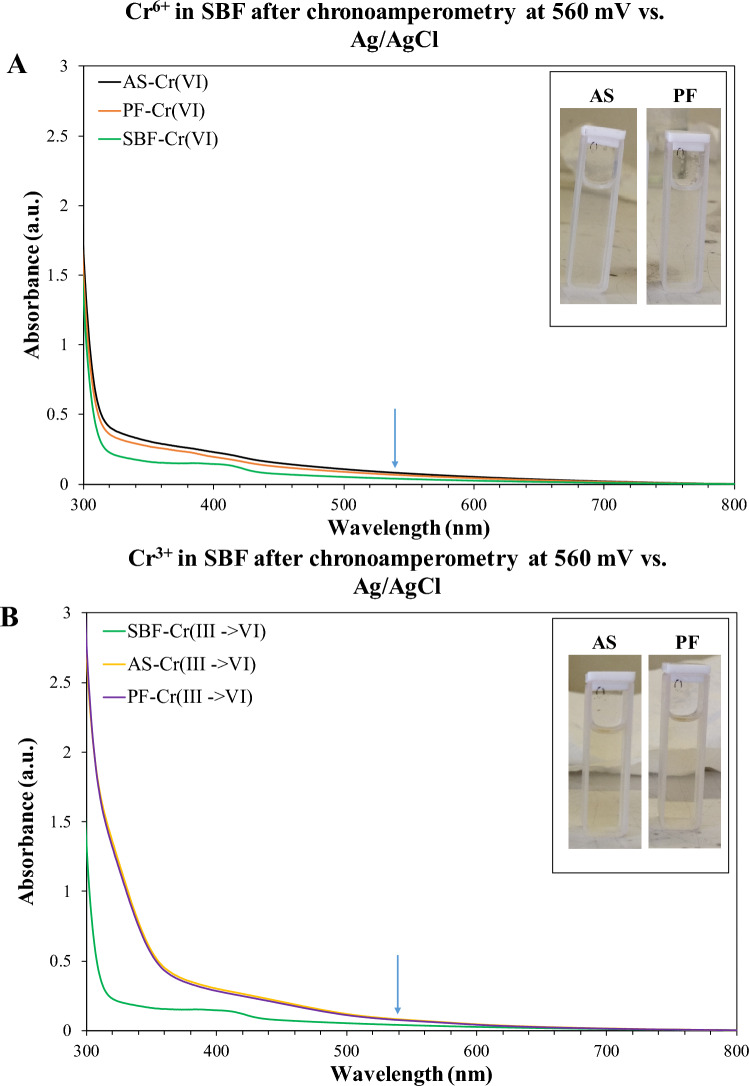
UV/vis spectra of the electrolytes after chronoamperometry of the AS alloy and PF alloy at 560 mV vs. Ag/AgCl (transpassive s tage 3). **A** Detection of Cr^6+^ in the electrolyte; **B** Detection of Cr^3+^ (that reacted with DPC to produce Cr^6+^) in the electrolyte; blue arrow: the absorbance wavelength of Cr^6+^. The spectrum of SBF is also included as a reference spectrum. Inset: The solutions after reaction with DPC

Figure 7 illustrates SEM, surface and cross-sectional, micrographs of AS and PF specimens after cyclic polarization (Fig. 7A–D) and surface micrographs of AS and PF specimens after chronoamperometry (Fig. 7E, F). The magnifications are relatively low for a general view. Both AS and PF specimens seem almost intact of corrosion after cyclic polarization. The free surfaces after chronoamperometry at 560 mV and 300 mV vs. Ag/AgCl show dispersed deposited particles but do not show any signs of pitting. EDX analysis revealed the compositions of three main types of deposited particles: opaque dark contrast flakes consisting of Co–Cr–C–Si–Mo–O (in yellow circles in Fig. 7E, F), rosette-like particles, which are coagulates of finer particles and consist of Co–Cr–Mo–Cl–C–Si or Co–Cr–Na–Cl–Mo–Si (yellow arrows in Fig. 7F), and NaCl crystals (white arrow in Fig. 7F). Figure 8A presents representative point EDX spectra from a rosette-like particle after chronoamperometry of AS alloy at 560 mV vs. Ag/AgCl, whereas Fig. 8B presents representative point EDX spectra from a rosette-like and a flake surface deposition of PF alloy after chronoamperometry at 300 mV vs. Ag/AgCl, in SBF, at 37 °C. The electrolytes after chronoamperometry testing of the AS and PF alloys at 560 mV vs. Ag/AgCl were analyzed for Cr^6+^ and Cr^3+^ using the diphenylcarbazide method, as described in the Materials and Methods section. Figure 9 illustrates the UV/vis spectra of the solutions after chronoamperometry testing at 560 mV vs. Ag/AgCl (a potential in passivity breakdown stage 3) of the AS and PF alloys. The spectrum of the as-prepared SBF solution is also included in Fig. 9 as a reference spectrum. The insets include the solutions after the reaction with DPC (in quartz cuvettes), which were subjected to absorbance measurements. None of the solutions were colored purple, whereas the characteristic peak at 540 nm is not present in any spectrum, suggesting the absence of Cr^6+^ and Cr^3+^ from the solutions.

**Fig. 10:**
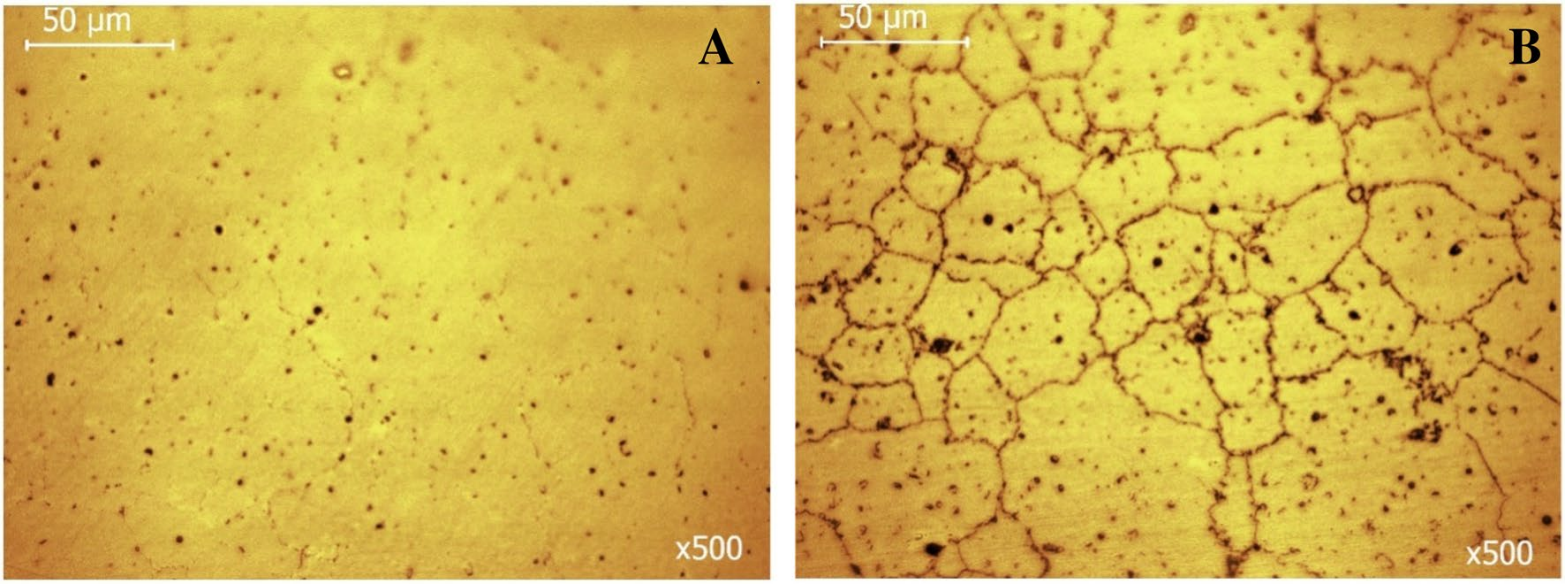
The surface state of the few replicates that underwent intergranular corrosion in SBF at 37 °C (AS: 2 out of 10, PF: 3 out of 10). **A** AS; **B** PF

Figure 10 shows the surface state of specimens, the cyclic voltammograms of which formed a clockwise hysteresis. Intergranular corrosion is revealed, which is more intensive in the PF specimen.

**Fig. 11:**
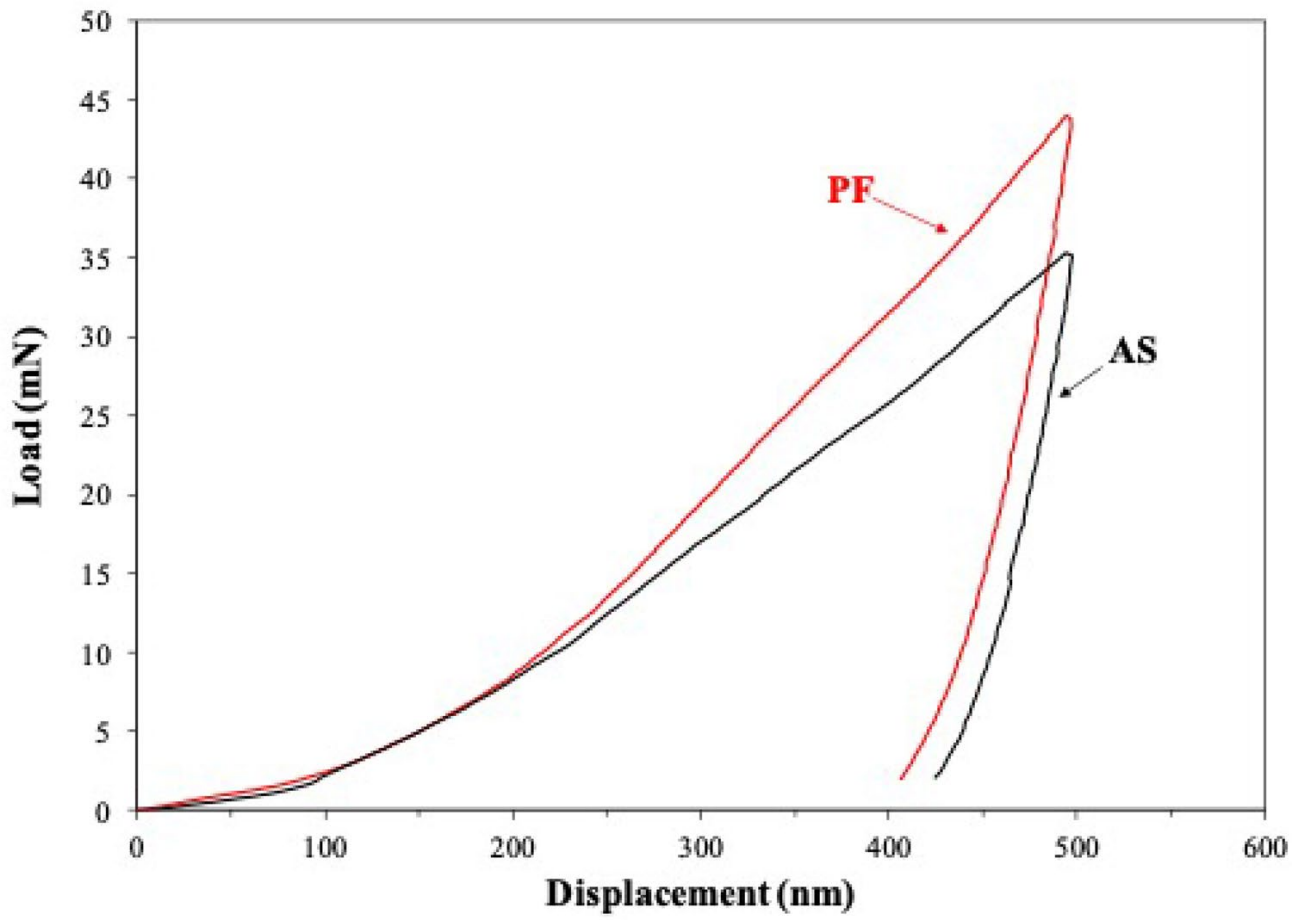
Representative load–displacement curves of AS Co–Cr–Mo and PF Co–Cr–Mo

Representative load–displacement curves from the nano-indentation tests are illustrated in Fig. 11. Figure 11 shows that in the case of PF Co–Cr–Mo, a greater load is needed to attain the same maximum displacement as in AS Co–Cr–Mo. Also, the unloading of the PF alloy has led to a slightly lower residual displacement (i.e., final depth of the contact impression) than that of the AS alloy. Table 3 lists the mean values and respective standard deviations of the indentation properties along with the p values. The PF group manifested greater microhardness, nanoindentation hardness, indentation modulus and true hardness, as compared to the AS group. It is important to note that although the PF Co–Cr–Mo specimens exhibited higher values compared to the AS Co–Cr–Mo specimens, only the indentation modulus and true hardness showed statistically significant differences (p = 0.0009 and 0.006, respectively).

**Table 3:** Microhardness, nanoindentation values and statistical significance between the subgroups (p ≤ 0.05 was considered significant)

	Microhardness Tester	Instrumented nano-indentation testing		
	Microhardness (HV)	Indentation hardness, Hit (GPa)	Indentation modulus, Eit (GPa)	True hardness (Hit/Eit)
Co–Cr–Mo_AS	285 ± 8	6.2 ± 0.7	251 ± 28	0.024 ± 0.003
Co–Cr–Mo_PF	323 ± 24	7.4 ± 0.5	275 ± 25	0.027 ± 0.003
p	1.23	3.34	0.0009	0.006

## Discussion

In this study, the effect of simulating porcelain firing cycles on the microstructure, corrosion behavior, and mechanical properties of a Co–Cr–Mo alloy produced by the MSM technique was investigated. According to the results of this in vitro study, the null hypothesis must be rejected, as significant differences were found in the mechanical properties tested.

The XRD pattern of the Co–Cr–Mo alloy (Fig. 1) reveals the presence of the two allotropes of Co, namely γ-Co, which is stable at high temperatures, and ε-Co, which is stable at low temperatures. The absence of ε-Co from the initial powder suggests that the ε-phase was formed during the MSM process. It is well established that the fcc-to-hcp transformation (also known as the ε-martensite transformation) in Co-based alloys can occur via three routes: athermally (by quenching from temperatures where the γ-phase is stable), isothermally (by aging in the temperature range of 650–950 °C) or by plastic strain inducing [34, 35]. In the case of the AS alloy, plastic strain induced by MSM and/or active recrystallization during sintering [14] seem the most likely causes for the martensitic transformation. Figure 1 and calculation of the martensite amount in the alloys according to Eq. (1) suggest a greater martensite presence in the PF alloy as compared to the AS alloy, most likely due to aging at the temperatures of the thermal cycling (as it will be justified in the following paragraphs). The martensitic transformation in Co–Cr–Mo alloys is quite sluggish [4], which justifies the predominance of fcc Co in both AS and PF Co–Cr–Mo.

The firing cycles also led to an increase in the carbide presence according to the XRD pattern of the PF alloy. XRD also detected the presence of SiO_2_, which will be discussed in the next paragraphs.

The microstructures of the AS and PF specimens are displayed in Fig. 2. The close similarity of the AS and PF microstructures is in compatibility with previous studies in cast, SLM and soft milled Co–Cr alloys [5, 19, 36]. The slight grain coarsening in the PF alloy may be attributed to annealing-grain growth mechanisms during simulating porcelain firing**.**

In context with the XRD findings, the increased presence of Mo and Cr along the grain boundaries (Fig. 3), which are well-known carbide stabilizing elements, suggests that the grain boundaries are delineated by carbide particles of (Cr,Mo)_23_C_6_, (Cr,Mo)_7_C_3_ and (Mo,Cr)C stoichiometries. The very fine carbide size, the intergranular presence and the detection of martensite (ε-phase) suggest the predominance of secondary M_23_C_6_ carbide in compatibility with Fig. 1. This is associated with the destabilization of the γ-matrix towards ε-phase formation and M_23_C_6_ precipitation during sintering and post-firing [37]. In fact, the heat treatment that simulated porcelain firing intensified both the martensitic transformation and the secondary carbide precipitation, which reflect on the diffractogram of the PF alloy in Fig. 1. The increased precipitation of secondary carbides during the firing cycles caused the depletion of fcc-Co (γ-Co) from Cr and Mo resulting in an increase of the M_s_ (“martensite start”) temperature, so that the unstable austenitic phase (γ-Co) transformed into martensite (ε-Co) upon cooling [38]. The increased precipitation of carbides due to the firing cycles also reflects on the thicker grain boundaries in Fig. 3C as compared with Fig. 3A.

The porosity shown in Fig. 2 has also been observed in previous efforts of Co–Cr–Mo alloys prepared by soft milling [5, 10, 19–21]. It has been attributed to necking of the metallic powder and evaporation of the organic binder during sintering [5]. Figures 3 and 4 show that the majority of the pores is filled with Si-oxide. Their presence can be justified by the following consideration: The porosity generated through powder-metallurgy techniques provides the fastest short-circuit diffusion pathways in a material [39]. As such, the presence of silica in the pores is the result of diffusion of oxygen and silicon into the pores (facilitated by the high temperatures involved during milling) and their consequent inter-reaction. Not only is Si the lightest element in the alloy and therefore, the easiest to diffuse, but it also has the greatest affinity for oxygen [40].

Overall, the microstructure inspection in corroboration with the XRD findings, has highlighted features important for the corrosion and mechanical performance of the AS and PF alloys, such as filling of pores with Si-oxide, increased amounts of martensite and intergranular carbides in the PF alloy, as well as slightly coarsened grains in the PF alloy compared to the AS alloy. These features will further be discussed in context with the findings of the electrochemical and mechanical study.

Figure 5 compares the potentiodynamic polarization curves of the AS and PF specimens. Table 2 lists critical electrochemical values extracted from the polarization curves of the coatings. The very similar shapes of the polarization curves of the AS and PF specimens indicate similar corrosion mechanisms. The counterclockwise hysteresis loops of marked surface area suggest non-susceptibility to localized corrosion.

Sharp changes in the anodic curve gradients divide them into several stages noted in Fig. 5. Stage 1, the active stage, is governed by the anodic dissolution of Co [41]. In most anodic scans, no clear active to passive transition is discerned, in compatibility with Milošev et al. [42] Stage 2, the passive stage, follows, which is sustained for a substantial range of potentials. In both cases, true passivity has been attained (i_p_ << 0.1 mA/cm^2^). Passivity has been attained by the formation of a barrier surface film, consisting predominantly of Cr_2_O_3_ and Cr(OH)_3_ at lower anodic potentials and being enriched with minor amounts of CoO and MoO_3_ at higher anodic potentials [42]. Stage 2 ends at the breakdown potential (E_b_) and it is succeeded by stage 3, a stage characterized by a rapid current density increase. Stage 3 can be ascribed to the transpassive dissolution of the Cr_2_O_3_ barrier layer by reaction (2):

2 $${\text{Cr}}_{2} {\text{O}}_{3} + {\text{ 5H}}_{2} {\text{O }} \to {\text{ 2CrO}}_{4}^{{2} - } + {\text{ OH}}^+ + {\text{ 6e}}^-$$

in compatibility with previous studies on Co–Cr–Mo alloys [41]. Stage 3 is succeeded by stage 4, characterized by a current decrease suggesting secondary passivation. Current densities during stage 4are two orders of magnitude greater than those of passive stage 2, which signifies that the chromate-based surface film is much less protective than the barrier film in stage 2. Stage 4 is succeeded by stage 5, the stage of oxygen evolution, during which hydroxyl ions in the solution or water molecules are oxidized according to reactions (3) and/or (4), respectively:

3 $${\text{4OH}}^- \to {\text{ O}}_{2} + {\text{ 2H}}_{2} {\text{O }} + {\text{ 4e}}^- \left( {\text{alkaline solutions}} \right)$$

4 $${\text{2H}}_{2} {\text{O }} \to {\text{ 4H}}^+ + {\text{ O}}_{2} + {\text{ 4e}}^- \left( {\text{acidic or neutral solutions}} \right)$$

Here it should be noted that oxygen evolution should have a major contribution to the current increase in stage 3. This is drawn from the following considerations: (a) The values of E_b_ (Table 2) are quite higher than those of the potential of transpassive dissolution of Cr_2_O_3_ (~ 290 mV vs. Ag/AgCl at pH = 7.4, at 25 °C, though [43], whereas they are close to the potential of oxygen evolution (~ 600 mV vs. Ag/AgCl at pH = 7.4, at 25 °C, though [43]. (b) Alloying of Cr with Co can lower the overpotential necessary to drive the reaction of water oxidation, since Co is an efficient catalyst for electrochemical water oxidation especially in the presence of phosphate in neutral water, as in the case of the SBF solution [44]. (c) The hysteresis loops during stage 3 are counterclockwise implying a sufficient protection of the surface film. Bettini et al. [45], investigating the transpassive dissolution of a Co–Cr–Mo alloy in a phosphate buffered saline (PBS) solution of pH = 7.4, at r.t., concluded that water oxidation largely contributed to the current increase at potentials above 500 mV vs. Ag/AgCl.

As it has to be clarified whether passivity in stage 2 is true and whether transpassivity in stage 3 is due to Cr_2_O_3_ dissolution, chronoamperometry was carried out at 100 mV and 300 mV vs. Ag/AgCl (stage 2) and 560 mV vs. Ag/AgCl (stage 3). The potentiostatic curves at 100 and 300 mV vs. Ag/AgCl, in Fig. 6, have the shape of typical passive behavior, namely an exponential decay of current density relaxing at steady current density values of the 10^–5^ mA/cm^2^ order. This behavior signifies a fast build-up of a surface layer to a maimum thickness, which is further ahead maintained. The potentiostatic curves at 560 mV vs. Ag/AgCl also exhibit a sharp drop of current density within the first ~ 1.5 min of immersion in SBF, which implies the fast formation and growth of a surface film. However, after attainment of a minimum current density value, an increase in current occurs, which is very subtle in the case of AS alloy (lasting for ~ 7.5 min) and more rapid in the case of PF alloy (lasting for ~ 11 min). Thereafter, a trend of a very slow increase in the current density accompanied by light (but occasionally sharp) fluctuations is sustained to the end of the test. The current densities attained after 2 h of testing are 0.013 mA/cm^2^ (AS) and 0.010 mA/cm^2^ (PF). The above trend indicates an anodic activity which, however, is too sluggish to disrupt the passivity. Therefore, and in conjunction with the low current densities and the counterclockwise hysteresis through stage 3, in Fig. 5, the postulation that stage 3 is rather owing to oxygen evolution and not due to Cr_2_O_3_-based film breakdown is enhanced. Nevertheless, the transpassivity nature will further be discussed after a few paragraphs in context with SEM inspection and Cr^6+^/Cr^3+^ colorimetric/spectrophotometric analysis.

In both groups (AS, PF), similar electrochemical values (critical potentials and current densities) were determined, as shown in Table 2. The corrosion potential values are essentially the same, reflecting the same phases composing the AS and PF alloys, as seen in Fig. 1. The very low i_corr_ values and the derived corrosion rates show the high resistance of both states to general corrosion. A slightly lower corrosion rate for the PF alloy cannot be confirmed, since the difference falls within standard deviation. Nevertheless, it could be justified by the slightly coarser grain size as compared to the AS alloy, i.e., less specific surface of grain boundaries, where corrosion cells between the noble carbides and the adjacent metallic matrix could be generated, and by the increased presence of hcp-Co, which is more stable than fcc-Co at low temperatures.

It seems that the presence of the Si-oxide dispersoids has benefited the corrosion resistance, as they have filled the pores and, hence, obstructed the penetration of the electrolyte in the inner parts of the specimens. However, it should be noted that a limited number of replicates presented clockwise hysteresis of small surface area, suggesting the occurrence of localized corrosion (AB: 2 out of 10, and AF: 3 out of 10).

Figure 7 illustrates SEM surface (Fig. 7A, B) and cross-sectional (Fig. 7C, D) micrographs of AS and PF specimens after cyclic polarization. In both cases, a fine surface state is manifested (Fig. 7A, B) without any signs of corrosion progress towards the interior of the specimens (Fig. 7C, D). Figure 7E, F manifest the fine surface state after chronoamperometry in traspassive stage 3 and passive stage 2, respectively. The dispersed particles in the surface (Fig. 7E, F) are products of interaction between the alloy and SBF, as seen in Fig. 8. The good surface state after chronoamperometry in transpassive stage 3 (Fig. 7E) confirms that breakdown is rather owing to O_2_ evolution than Cr_2_O_3_ oxidation. The latter was double-checked by the colorless, fully transparent solutions of the DPC method showing that there was not any Cr^6+^ (or even Cr^3+^ oxidized to Cr^6+^) to react with 1,5-DPC leading to a purple colorization and by the absence of any peak at the wavelength of 540 nm in the UV/vis spectra of Fig. 9.

Figure 10 presents the surface state of two specimens, the cyclic polarization of which led to clockwise hysteresis. Intergranular attack is the main form of corrosion owing to the presence of noble carbides along grain boundaries. Intergranular attack is more intensive in the PF alloy due to the increased presence of carbides, according to the XRD results (Fig. 1).

In conclusion, the counterclockwise loops upon cyclic polarization along with the sustainable regime of true passivity and the microstructural state after corrosion of the soft-milled alloys imply the high resistance of the alloys to localized corrosion. Breakdown of passivity has been attributed to the oxygen evolution reaction. The very low corrosion current densities (order of 10^–4^ mA/cm^2^) imply the high resistance of the alloys to uniform corrosion, to an extent realized by the filling of pores with Si-oxide. The total absence of any Cr^3+^ or Cr^6+^ in the solution after chronoamperometry in the transpassive stage is of paramount clinical importance. Therefore, it is suggested that MSM Co–Cr–Mo in both the AS and PF states, is resistant to corrosion in SBF solution and can, thus, be employed for biomedical applications from a corrosion standpoint.

Table 3 compares the mechanical properties of the AS and PF alloy determined by indentation. The PF specimens present higher microhardness compared to the AS specimens. This is attributed to the increased presence of ε-Co and carbides. ε-Co has lower number of independent slip systems than γ-Co and, hence, less ductility but greater strength [46]. At the same time, fine carbide precipitation at grain boundaries is considered the main strengthening mechanism in Co–Cr alloys [21]. Both values are comparable with values reported in literature for CAD/CAM milled and CAD/CAM milled/conventionally sintered Co–Cr–Mo alloys [47, 48]. Mengucci et al. [49] have attributed the high hardness of Direct Metal Laser sintered Co–Cr–Mo dental alloys to the formation of an intricate network of ε-Co (hcp) lamellae in the γ-Co (fcc) matrix.

Regarding the nano-indentation measurements, the load–displacement curves in Fig. 11 show that for PF Co–Cr–Mo, a greater load is needed to attain the maximum displacement of 500 nm as compared to AS Co–Cr–Mo, which reflects a higher strength. On the other hand, the lightly lower residual displacement of the PF specimens suggests a lower ability of plastic deformation in comparison with the AS specimens. The residual displacement of both alloys is less than 450 nm, which shows that both alloys have undergone small plastic deformation [50].

The indentation modulus (Eit) values of the Co–Cr–Mo specimens are comparable with those reported in literature and moderately to notably higher than those of other milled and soft-milled Co–Cr alloys, which, however, were measured by tensile straining [10, 51]. They are also higher than the indentation modulus values of cast and SLM alloys by a few decades of GPa [52], and very close to the indentation modulus of a Co–Cr implant alloy used as a substrate to thermally sprayed hydroxyapatite [50]. The indentation modulus values herein obtained exceed the ASTM F75 requirement for the elastic modulus of unfinished Co-28Cr-6Mo investment castings for surgical implant applications (210–250 MPa).

The nano-indentation hardness (Hit) values are comparable with those of cast and SLM Co–Cr alloys obtained by nano-indentation [52]. They are also very close to the Hit values of a Co–Cr implant alloy used as a substrate to thermally sprayed hydroxyapatite [50]. In addition, these values are lower (by about 0.4–1.6 GPa) than the Hit values of TIG-surface treated Co–Cr–Mo [4].

The nano-indentation measurements confirmed a modest superiority of the PF specimens in terms of (a) greater resistance to reversible deformation (higher indentation modulus), (b) greater resistance to irreversible and reversible deformation (higher indentation hardness), and (c) greater resistance to plastic deformation (higher true hardness, namely higher Hit/Eit ratio). Regarding (a), it should be noted that the indentation modulus could be slightly different from the Young’s modulus since it is a weighted average of the elastic properties in a certain sample volume whereas the Young’s modulus is directional. Nonetheless, for isotropic materials, both values should be equal [53]. Regarding (b), the indentation hardness is a hybrid measure of a material’s resistance to irreversible and reversible deformation, so that a change in the indentation modulus will also affect the indentation hardness [54]. Regarding (c), the Hit/Eit ratio can be used to determine the resistance to irreversible deformation, and represents the energy required to create a unit volume of irreversible impression [55]. It should be noted that the Hit/Eit ratio may be interpreted as an ‘elasticity index’: Large values suggest a predominance of reversible deformation, whilst small values, as in the present cases, suggest a predominance of irreversible deformation [55]. Hence, in the present cases, the Hit/Eit ratio is also an index of the yield strength of the material [54]. Higher true hardness also indicates higher wear resistance [52].

In conclusion, as regards the mechanical property evaluation, PF has led to an increase in the hardness, yield strength and indentation modulus of the AS alloy, manly attributed to the increase in the martensite and intergranular carbide presence. All the measured values are comparable with values reported in literature. Furthermore, they satisfy the mechanical property requirements (modulus of elasticity greater than 200 GPa and microhardness in the range of 200–350 HV) [1].

As for the clinical significance of the aforementioned results, both states (AS and PF) meet the requirements for corrosion resistance (high resistance to general and localized corrosion, true passivity, as well as not any Cr^6+^ formation upon passivity breakdown) and the just mentioned mechanical property requirements.

The mechanical properties studied are of paramount importance for the clinical performance of fixed metal-ceramic dental restorations [56]. As shown in Table 3, the indentation modulus and indentation hardness values are higher for the PF Co–Cr–Mo specimens, which is desirable because with this increase: (i) the stresses exerted on the porcelain are reduced, (ii) the deformation of the metal substrate during the cooling of the dental porcelain is reduced, and (iii) the elastic deformation of the metal substrate during masticatory loading is suppressed [6]. The three advantages mentioned above result in the brittle ceramic material being protected from fractures, which enables the design of a prosthetic restoration made of Co–Cr–Mo with a minimal thickness. This significantly improves aesthetic results, as less grinding of the natural tooth and using up of more space in the process of covering the metal substrate with a ceramic material, are achieved [1, 6]. After the simulating porcelain firing procedure, the Hit/Eit ratio also increased (from 0.024 ± 0.003 to 0.027 ± 0.003). Considering that the Hit/Eit ratio is an index of the yield strength of the material [54], the relatively high values of this property for the PF Co–Cr–Mo specimens will better protect the metal-ceramic system against the onset of plastic deformation (or irreversible deformation), as compared to the AS Co–Cr–Mo alloy [1, 55]. Bearing in mind that a ceramic material cannot undergo plastic deformation, in case such deformation occurs in the metal-ceramic restorations, fracture and detachment of the aesthetic ceramic material during repeated chewing cycles, especially in the thin neck areas, will be observed [6]. As for the microhardness, the values of this property should preferably be between ~ 200–350 HV, since a hard alloy will cause difficulty in polishing, which may lead to microbial plate accumulation, which in turn may negatively affect the health of the abutment tooth [6, 56].

In summary, the corrosion and mechanical responses of the Co–Cr–Mo alloys herein studied not only qualify the MSM technique and the porcelain firing process (used in the fabrication of metal-ceramic restorations) as trustworthy/safe methods for the fabrication of Co–Cr–Mo substrates for metal-ceramic dental restorations, but they also underline the importance of post heat-treating (porcelain firing process) in improving the mechanical behavior of Co–Cr–Mo alloys under the dynamically changing loads in the oral cavity without jeopardizing their fine corrosion performance.

## Conclusions

The following conclusions are drawn from the comparative study of as-sintered (AS) and post-fired (PF) specimens of dental Co–Cr–Mo alloy prepared by the Metal Soft Milling (MSM) technique:

- Besides a slight grain coarsening and a modest increase in the hcp-Co content, simulating porcelain firing did not cause any marked difference in the microstructure of the AB alloy. Both, AS and PF alloys consisted of a Co-based (a mixture of fcc-Co and hcp-Co) matrix with a diffuse distribution of pores (most of which were filled with Si-oxide) and a fine carbide (mostly M_23_C_6_) intergranular precipitation.
- Despite the extensive distribution of pores (filled with Si-oxide though), both alloys showed very low rates of uniform corrosion (order of 10^–4^ mA/cm^2^ or 10^–3^ mm/y) and high resistances to localized corrosion, in SBF at 37 °C. Breakdown of passivity at overpotentials close to 500 mV vs. Ag/AgCl was not due to Cr_2_O_3_ transpassive dissolution but due to O_2_ evolution. Hence, it must be underlined that simulating porcelain firing procedure did not jeopardize the corrosion properties of the initial AS alloy.
- Simulating porcelain firing led to an increase in the microhardness (from 285 ± 8 to 323 ± 24 HV), nano-indentation hardness (from 6.2 ± 0.7 to 7.4 ± 0.5 GPa), nano-indentation modulus (from 251 ± 28 to 275 ± 25) and true hardness (from 0.024 ± 0.003 to 0.027 ± 0.003), namely greater resistance to both irreversible and reversible deformation. The indentation modulus and true hardness showed significant differences, p = 0.0009 and 0.006, respectively.
- The above findings not only qualify both the MSM technique and the simulating porcelain firing process (used in the fabrication of metal-ceramic restorations) as trustworthy/safe methods for the production of Co–Cr–Mo substrates for metal-ceramic dental prosthetic restorations, but they also emphasize the importance of the simulating porcelain firing process in improving the mechanical behavior of the Co–Cr–Mo alloys under the dynamically changing loads on the oral cavity without jeopardizing their corrosion resistance.
